# Supplementary material for: The C1473G Mutation in the Mouse Tph2 Gene: From Molecular Mechanism to Biological Consequences
Source: Biomolecules. 2025 Mar 21;15(4):461. doi: 10.3390/biom15040461 (PMC12024906; doi:10.3390/biom15040461)

<u>TPH2 and GAPDH western blots (Fig. 6)</u>

Groups:

C – 1473C mice

G – 1473G mice

0 – test probe, not included in the results

TPH2 protein. Calculated molecular weight – 56 kDa.

Probe groups: G, C, G, C, G, C, G, C, G, 0, G, C, G, C, G, C, G, G

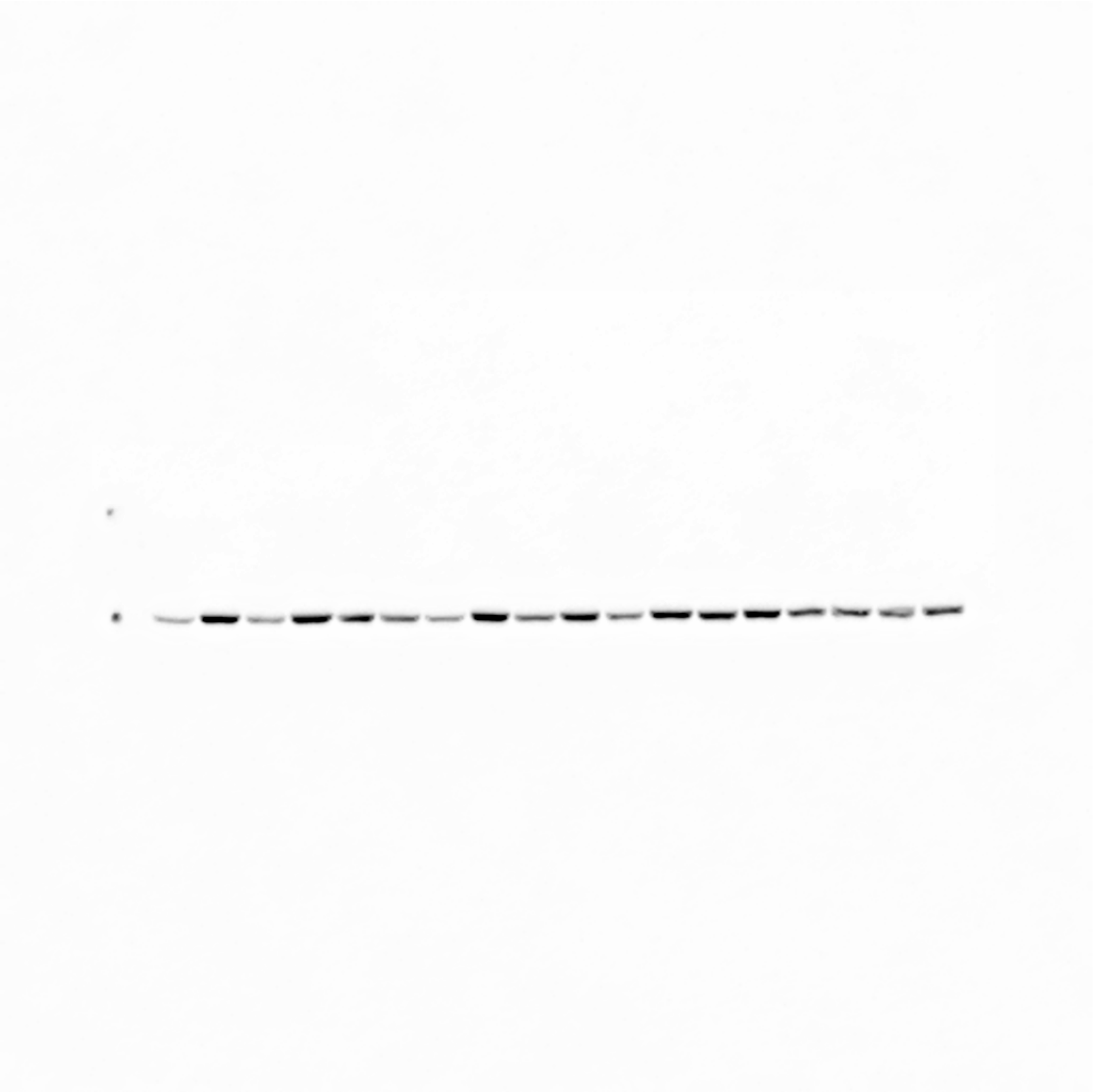

GAPDH protein. Calculated molecular weight – 37 kDa. Markers: 37 kDa.

Probe groups: G, C, G, C, G, C, G, C, G, 0, G, C, G, C, G, C, G, G

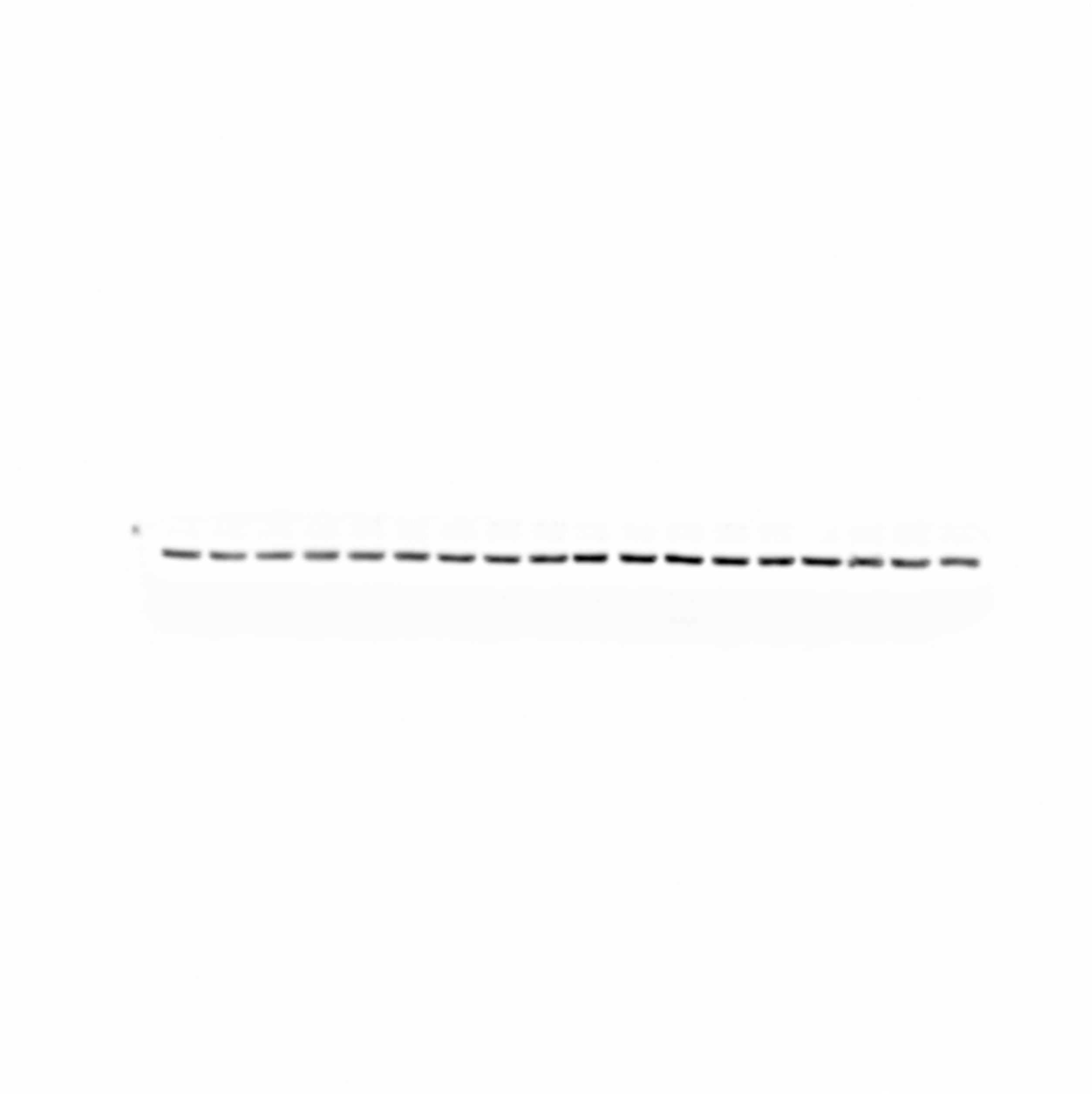

Supplement: Supplementary file 1 [file biomolecules-15-00461-s001.zip › biomolecules-3421224-supplementary.pdf]
